# Sodium glucose co-transporter-2 inhibitors in intensive care unit patients with type 2 diabetes: a pilot case control study

**DOI:** 10.1186/s13054-023-04481-y

**Published:** 2023-05-16

**Authors:** Johan Mårtensson, Salvatore Lucio Cutuli, Eduardo A. Osawa, Fumitaka Yanase, Lisa Toh, Luca Cioccari, Nora Luethi, Akinori Maeda, Rinaldo Bellomo

**Affiliations:** 1 Department of Physiology and Pharmacology, Section of Anaesthesia and Intensive Care, Karolinska Institutet, Stockholm, Sweden; 2 Department of Perioperative Medicine and Intensive Care, Karolinska University Hospital, 171 76 Stockholm, Sweden; 3 Dipartimento di Scienze dell’Emergenza, Anestesiologiche e della Rianimazione, Fondazione Policlinico Universitario Agostino Gemelli IRCCS, Rome, Italy; 4 Dipartimento di Scienze Biotecnologiche di Base, Cliniche Intensivologiche e Perioperatorie, Università Cattolica del Sacro Cuore, L.Go F. Vito 1, 00168 Rome, Italy; 5 Cardiology Intensive Care Unit, Hospital DF-Star, Brasília, Brazil; 6 Department of Intensive Care, Austin Health, Melbourne, VIC Australia; 7 Australian and New Zealand Intensive Care Research Centre, Monash University, Melbourne, VIC Australia; 8 Department of Intensive Care Medicine, Kantonsspital Aarau, Aarau, Switzerland; 9 Department of Emergency and Critical Care Medicine, The University of Tokyo Hospital, Tokyo, Japan; 10 Department of Critical Care, Melbourne University, Melbourne, VIC Australia; 11 Department of Intensive Care, Royal Melbourne Hospital, Melbourne, Australia

**Keywords:** SGLT2 inhibitor, Empagliflozin, Intensive care, Diabetes

## Abstract

**Background:**

Sodium glucose co-transporter-2 (SGLT2) inhibitors improve long-term cardiovascular and renal outcomes in individuals with type 2 diabetes. However, the safety of SGLT2 inhibitors in ICU patients with type 2 diabetes is uncertain. We aimed to perform a pilot study to assess the relationship between empagliflozin therapy and biochemical, and clinical outcomes in such patients.

**Methods:**

We included 18 ICU patients with type 2 diabetes receiving empagliflozin (10 mg daily) and insulin to target glucose range of 10–14 mmol/l according to our liberal glucose control protocol for patients with diabetes (treatment group). Treatment group patients were matched on age, glycated hemoglobin A1c, and ICU duration with 72 ICU patients with type 2 diabetes exposed to the same target glucose range but who did not receive empagliflozin (control group). We compared changes in electrolyte and acid–base parameters, hypoglycemia, ketoacidosis, worsening kidney function, urine culture findings, and hospital mortality between the groups.

**Results:**

Median (IQR) maximum increase in sodium and chloride levels were 3 (1–10) mmol/l and 3 (2–8) mmol/l in the control group and 9 (3–12) mmol/l and 8 (3–10) mmol/l in the treatment group (*P* = 0.045 for sodium, *P* = 0.059 for chloride). We observed no differences in strong ion difference, pH or base excess. Overall, 6% developed hypoglycemia in each group. No patient in the treatment group and one patient in the control group developed ketoacidosis. Worsening kidney function occurred in 18% and 29% of treatment and control group patients, respectively (*P* = 0.54). Urine cultures were positive in 22% of treatment group patients and 13% of control group patients (*P* = 0.28). Overall, 17% of treatment group patients and 19% of control group patients died in hospital (*P* = 0.79).

**Conclusions:**

In our pilot study of ICU patients with type 2 diabetes, empagliflozin therapy was associated with increases in sodium and chloride levels but was not significantly associated with acid–base changes, hypoglycemia, ketoacidosis, worsening kidney function, bacteriuria, or mortality.

**Supplementary Information:**

The online version contains supplementary material available at 10.1186/s13054-023-04481-y.

## Introduction

Data from large randomized controlled trials have shown that sodium glucose co-transporter-2 (SGLT2) inhibitors attenuate cardiovascular and kidney disease progression regardless of the presence or absence of diabetes [1]. In addition, in patients with chronic kidney disease and marked albuminuria, treatment with the SGLT2 inhibitor dapagliflozin also reduced the risk of abrupt declines in kidney function as defined by a doubling of serum creatinine between two subsequent study visits (median time-interval 100 days) [2].

However, the safety and efficacy of SGLT2 inhibitors in critically ill patients treated in the intensive care unit (ICU) has never been assessed. Concerns include observed associations between SGLT2 inhibitors and increased risk of normoglycemic ketoacidosis [3], urinary tract infections [4], electrolyte and acid–base disturbances [5], and early decline in glomerular filtration rate triggered by tubuloglomerular activation and decreased intraglomerular pressure [6].

On the other hand, in non-critically ill patients with cardiometabolic risk factors who were hospitalized with COVID-19, dapagliflozin therapy resulted in a numerical reduction in organ failure, including acute kidney injury, renal replacement therapy and death within 30 days compared with placebo [7]. Although differences were not statistically significant, these data suggest that SGLT2 inhibitors may be safely used in acutely ill patients.

ICU patients with type 2 diabetes experience acute-on-chronic insulin resistance, and typically require high insulin doses to achieve target blood glucose levels [8]. Unfortunately, insulin therapy increases the risk of excessive glucose fluctuations and hypoglycemia, and these risks are exacerbated in critically ill patients with diabetes [9, 10]. Previous studies suggest that large glucose fluctuations and hypoglycemia provide mechanistic links between insulin therapy and adverse clinical outcomes [11, 12]. Adding SGLT2 inhibitors has the potential to reduce insulin requirements. However, reduced insulin delivery could result in relative insulin deficiency and ketoacidosis.

Given the above concerns and lack of data in the ICU setting, we conducted a pilot case–control study in ICU patients with type 2 diabetes receiving insulin aiming to evaluate whether the potential to reduce insulin requirements using the SGLT2 inhibitor empagliflozin outweighed the risk of ketoacidosis. In addition, we aimed to assess the relationship of empagliflozin therapy with biochemical, renal, infectious, and exploratory clinical outcomes.

## Material and methods

This study was approved by the ethics committee at Austin Hospital, Melbourne, Australia (approval HREC No. LNR/14/Austin/487) with a waiver of informed consent.

### Patients

In this pilot, matched case–control study, we included adult (18 years or older) patients with type 2 diabetes admitted to ICU at Austin Hospital, Melbourne, Australia in whom insulin infusion was commenced within the previous 24 h. Patients receiving chronic empagliflozin therapy, pregnant patients and patients with a do not resuscitate order were not eligible for inclusion. Included patients were prescribed 10 mg empagliflozin per day until discharge from ICU. Enrolment was stopped after inclusion of 18 patients due to the warning issued by FDA that SGLT2 inhibitors increase the risk of Fournier’s gangrene [13]. Here, we present the data for these 18 patients. All patients were managed according to our liberal glucose control protocol [14]. According to the protocol, intravenous insulin infusion was initiated when glucose exceeded 14 mmol/L and adjusted to a target level between 10 and 14 mmol/L while in the ICU. Hourly blood glucose measurements were recommended to guide insulin therapy (Additional file 1: Fig. S1). We selected control group patients from an existing, prospectively collected, database including 350 ICU patients with diabetes treated according to the same glucose control protocol but who did not receive SGLT2 inhibitors in the ICU [14]. From this database, we excluded patients with type 1 diabetes, patients with end-stage renal disease, and patients without available HbA1c or admission creatinine. The remaining 269 patients were matched (four controls per case) using coarsened exact matching on the following variables: age (category < 50 years, 50–60 years, > 60 years), HbA1c (above or below 7%), and ICU length of stay (category 0–2 days, 2–5 days, 5–10 days, > 10 days). No patient in the control group or in the treatment group received noninsulin glucose-lowering drugs (except empagliflozin in the treatment group) while in the ICU.

### Data collection

In control group and treatment group patients, we prospectively recorded demographic data, ICU admission characteristics, pre-ICU diabetes treatment, admission glycated hemoglobin A1c (HbA1c), daily insulin administration, urine output and renal replacement therapy use in the ICU, and routine blood gas data obtained in the ICU. We measured glucose, sodium, chloride, potassium, base excess, pH and creatinine in arterial or venous blood using the Radiometer ABL825 blood gas analyser (Radiometer Medical A/S, Brønshøj, Denmark). Admission HbA1c was analyzed in routine blood samples using the COBAS INTEGRA 800 analyser (Roche Diagnostics, Indianapolis, IN). In treatment group patients we measured blood β-hydroxybutyrate each morning (at the time of empagliflozin administration) using the Freestyle Optium Xceed point-of-care meter (Abbott Diabetes Care Inc., Maidenhead, UK). In the control group, blood β-hydroxybutyrate was measured as soon as possible after ICU admission and each morning thereafter in a subgroup of patients [15].

### Outcomes

We assessed biochemical, renal, infectious, and exploratory clinical outcomes. Biochemical outcomes included maximum increase in blood electrolyte levels (sodium, chloride, and potassium) and acid–base parameters (abbreviated strong ion difference [aSID], pH, and base excess), hypoglycemia occurrence (blood glucose level < 4 mmol/l), and occurrence of ketosis (β-hydroxybutyrate level ≥ 0.6 mmol/l) and ketoacidosis (beta-hydroxybutyrate level ≥ 3 mmol/l in combination with bicarbonate level < 15 mmol/l or pH < 7.3). Blood glucose level ≥ 11 mmol/l was not included in the definition of ketoacidosis since patients were treated according to a liberal glucose protocol and since empagliflozin may cause ketoacidosis despite normoglycemia. Renal outcomes included maximum increase in creatinine level, worsening kidney function (doubling of creatinine relative the first creatinine value in ICU [control group] or relative the most recent creatinine value before empagliflozin initiation [treatment group] and/or receipt of new renal replacement therapy), and the diuretic response to furosemide therapy (urine output to furosemide dose ratio). Infectious outcome included a positive urine culture. Exploratory clinical outcome included hospital mortality.

### Statistical analysis

Continuous variables were summarized as median (interquartile range [IQR]) and categorical variables as *n* (%). We compared continuous data using the Mann-Whitney U test. For categorical variables, we used the chi-squared test or the Fisher’s exact test. We used generalized linear mixed modeling with random intercept per individual and random slope over time to assess changes in urine output to furosemide dose ratio over time. The interaction between group and time was introduced in the mixed model to compare the change over time between groups. A two-sided *P* value < 0.05 was considered statistically significant in the analyses. We analyzed data using STATA version 16.1 (Stata Corp., College Station, TX, USA).

## Results

### Patients

The characteristics of the 72 control group patients and the 18 treatment group patients are compared in Table 1. The distribution of the matching variables (age, HbA1c and ICU length of stay) was similar in the two groups. As compared with control group patients, treatment group patients were more likely to have cardiovascular, renal and/or lung diseases and were more likely to be admitted from the emergency department. Treatment group patients were more likely to be admitted with sepsis whereas cardiovascular, gastrointestinal, and renal admission diagnoses were more common in the control group. A detailed list of pre-ICU diabetes therapy in treatment group patients is provided in Additional file 1: Table S1.

**Table 1:** Patient characteristics

Characteristic	Control group (*n* = 72)	Treatment group (*n* = 18)	*P* value
Age, years	66 (59–73)	60 (53–69)	0.15
HbA1c, %	7.7 (6.5–8.5)	7.7 (7.2–9.6)	0.38
ICU length of stay, days	6 (3–10)	8 (4–16)	0.14
Male sex	47/72 (65%)	14/18 (78%)	0.31
Body weight, kg^a^	87 (72–108)	94 (83–102)	0.31
Comorbidity			
Cardiovascular disease	7/72 (10%)	11/18 (61%)	< 0.001
Renal disease	0/72 (0%)	4/18 (22%)	< 0.001
Lung disease	9/72 (12%)	6/18 (33%)	0.034
Liver disease	9/72 (12%)	2/18 (11%)	0.87
Baseline creatinine, µmol/l	81 (62–142)	98 (88–101)	0.88
Diabetes therapy			0.93
Insulin only	12/72 (17%)	4/18 (22%)	
Insulin & noninsulin glucose-lowering drugs	17/72 (24%)	4/18 (22%)	
Noninsulin glucose-lowering drugs only	32/72 (44%)	8/18 (44%)	
Diet control only	11/72 (15%)	2/18 (11%)	
Location before ICU			< 0.001
Operating room	44/71 (62%)	4/18 (22%)	
Emergency department	5/71 (7%)	8/18 (44%)	
Ward	17/71 (24%)	5/18 (28%)	
Other hospital	5/71 (7%)	1/18 (6%)	
Admission diagnosis^b^			
Sepsis	4/72 (6%)	10/18 (56%)	< 0.001
Cardiovascular	37/72 (51%)	3/18 (17%)	0.008
Neurological	6/72 (8%)	2/18 (11%)	0.71
Gastrointestinal	14/72 (19%)	0/18 (0%)	0.042
Respiratory	7/72 (10%)	4/18 (22%)	0.15
Trauma	1/72 (1%)	1/18 (6%)	0.28
Renal	43/72 (60%)	0/18 (0%)	< 0.001
Hematological	2/72 (3%)	0/18 (0%)	0.47
Mechanical ventilation	53/72 (74%)	15/18 (83%)	0.39
Renal replacement therapy	3/72 (4%)	1/18 (6%)	0.80
Vasopressor infusion	29/72 (40%)	12/18 (67%)	0.044
APACHE III score	60 (42–77)	62 (46–71)	0.94

Data are presented as median (IQR) for continuous measures, and n/total number with data (%) for categorical measures

*HbA1c* glycated hemoglobin A1c, *ICU* intensive care unit, *APACHE* acute physiology and chronic health evaluation

^a^Data was missing in 5 patients in the control group

^b^Patients can have more than one admission diagnosis

### Empagliflozin therapy

In the treatment group, the first empagliflozin dose was administered after a median (IQR) of 33.9 (15.4–53.6) hours after ICU arrival. Empagliflozin was administered daily for a median of 5.5 (2.0–9.0) ICU days.

### Biochemical outcomes

The median (IQR) maximum absolute increase in blood sodium and chloride levels were 3 (1–10) mmol/l and 3 (2–8) mmol/l in the control group and 9 (3–12) mmol/l and 8 (3–10) mmol/l in the treatment group (*P* = 0.045 for sodium and *P* = 0.059 for chloride).

**Fig. 1:**
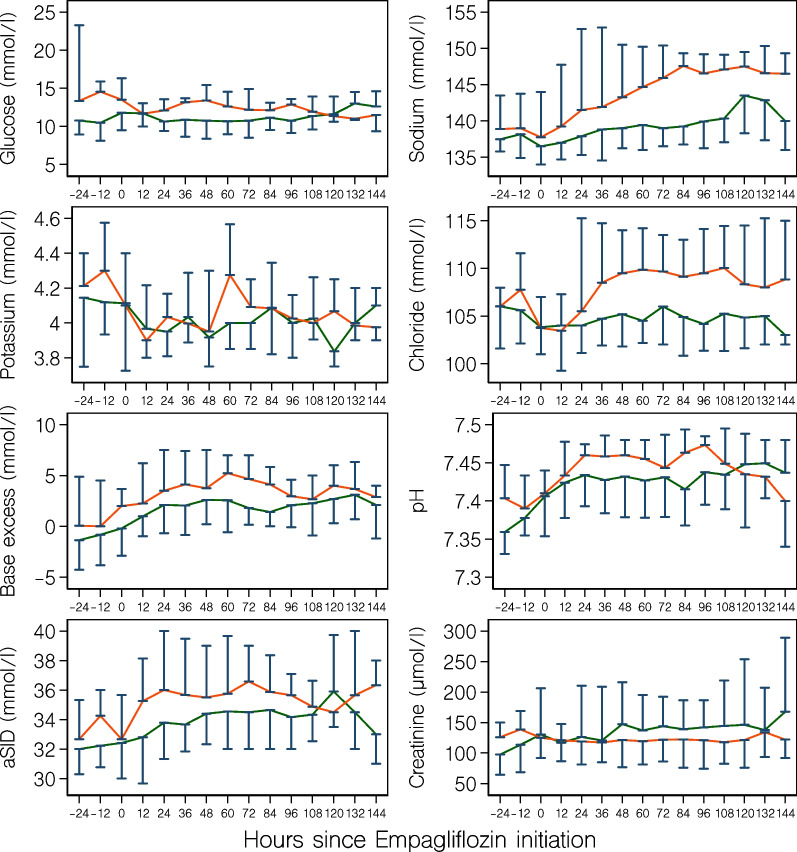
Median (IQR) blood gas parameters during 24 h before until 144 h after initiation of empagliflozin therapy (red curves). Minus 24 h data are the averages of the measurements obtained 36–24 h before therapy; minus 12 h data are the averages of the measurements obtained 24–12 h before therapy; zero hour data are the averages of the measurements obtained 12 h before therapy; twelve hour data are the averages obtained during the first 12 h after therapy initiation. In the control group (green lines), time zero was set at 34 h after ICU admission (corresponding to the median time between ICU admission and empagliflozin initiation in the treatment group). aSID, abbreviated strong ion difference

**Fig. 2:**
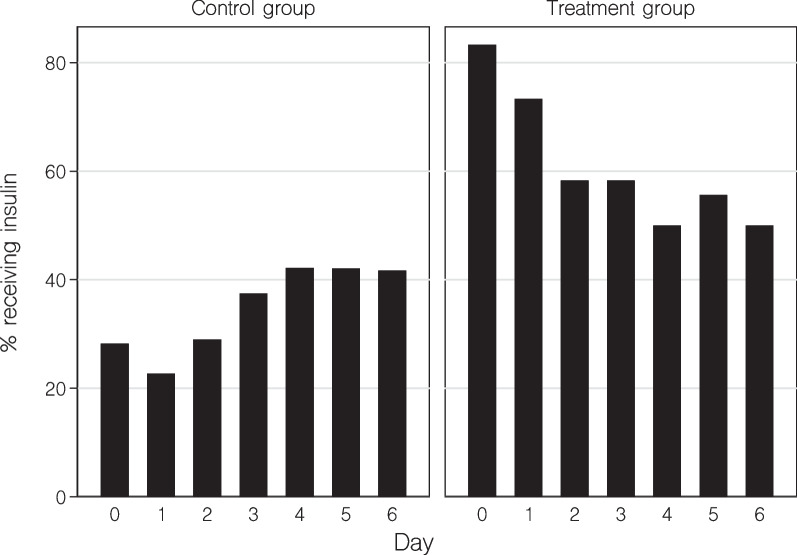
Percentage of control group patients receiving insulin (left panel) and percentage of empagliflozin treated patients receiving insulin (right panel) during the first week in ICU. In control group patients, day zero is the day of ICU arrival. In treatment group patients, day zero is the day when empagliflozin treatment was initiated

Median absolute changes in aSID, pH, and base excess were similar in the two groups (Table 2 and Fig. 1). The proportion of patients receiving insulin was higher in the treatment group (Fig. 2). However, while this proportion increased in the control group over time, a decrease was observed after initiation of empagliflozin. Blood glucose profiles were similar in the two groups (Fig. 1). Overall, four (6%) control group patients and 1 (6%) treatment group patient developed hypoglycemia (Table 2). Blood β-hydroxybutyrate levels were available in 15 out of 72 (21%) patients in the control group and in 16 out of 18 (89%) patients in the treatment group. A total of 93 and 102 blood ketone measurements were analyzed in the control group and treatment group, respectively. Most measurements we performed between 4 and 8 am (Additional file 1: Fig. S2). Approximately half of patients in each group developed some degree of ketosis. However, marked ketosis or ketoacidosis were rare events (Table 2).

**Table 2:** Outcomes

Outcome measure	Control group^a^ (*n* = 72)	Treatment group^b^ (*n* = 18)	*P* value
Maximum increase in blood electrolyte levels			
Sodium, mmol/l	3 (1–10)	9 (3–12)	0.04
Chloride, mmol/l	3 (2–8)	8 (3–10)	0.052
Potassium, mmol/l	0.9 (0.3–1.3)	0.7 (0.4–1.4)	0.79
Maximum increase in acid–base parameters			
aSID, mmol/l	5 (2–7)	6 (3–11)	0.26
pH	0.09 (0.05–0.2)	0.08 (0.05–0.13)	0.28
Base excess, mmol/l	5 (2–8)	4 (1–6)	0.35
Hypoglycemia (< 4 mmol/l)	4/72 (6%)	1/18 (6%)	> 0.99
Ketosis (Beta-hydroxybutyrate level)			
No ketosis (< 0.6 mmol/l)	7/15 (47%)	9/16 (56%)	0.72
Mild ketosis (0.6–1.5 mmol/l)	4/15 (27%)	1/16 (6%)	
Moderate ketosis (1.6–2.9 mmol/l)	1/15 (7%)	5/16 (31%)	
Marked ketosis (≥ 3 mmol/l)	3/15 (20%)	1/16 (6%)	
Ketoacidosis^c^	1/15 (7%)	0/16 (0%)	0.48
Renal outcomes			
Maximum increase in creatinine, µmol/l	34 (11–97)	21 (0–34)	0.17
Worsening kidney function^d, e^	20/69 (29%)	3/17 (18%)	0.54
Receipt of new renal replacement therapy^e^	13/69 (19%)	1/17 (6%)	0.28
Infectious outcome			
Positive urine culture (any pathogen)	9/72 (13%)	4/18 (22%)	0.28
Exploratory clinical outcome			
Hospital mortality	14/72 (19%)	3/18 (17%)	0.79

Values are median (IQR) or n (%)

*aSID* abbreviated strong ion difference

^a^Outcomes in the control group were assessed from ICU admission until ICU discharge

^b^Outcomes in the treatment group were assessed from empagliflozin initiation until ICU discharge

^c^Beta-hydroxybutyrate level ≥ 3 mmol/l in combination with bicarbonate level < 15 mmol/l or pH < 7.3

^d^Doubling of creatinine relative the first creatinine value in ICU (control group) or relative the most recent creatinine value before empagliflozin initiation (treatment group) or receipt of new renal replacement therapy

^e^Patients with renal replacement therapy on admission were excluded from the analysis

### Renal outcomes

**Fig. 3:**
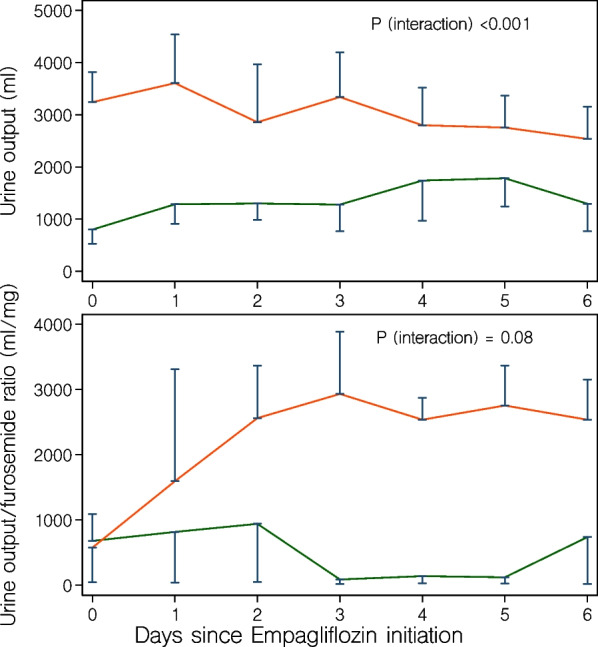
Median (IQR) total daily urine output (upper panel) and daily urine output to furosemide dose ratio (lower panel) during the first seven days since empagliflozin initiation (red lines). In the treatment group (red lines), day zero is the day of empagliflozin initiation. In the control group (green lines), day zero is the day of ICU arrival. Urine output to furosemide dose ratio was calculated by dividing the total daily urine output (ml) by the total amount (in mg) of furosemide administered on the same day. When no furosemide was administered on a single day, a zero dose was replaced by 1.0 mg in the denominator. *P* values represent the interaction between group and time on generalized linear mixed modeling

The median (IQR) maximum absolute increase in creatinine was 34 (11–97) µmol/l in the control group and 21 (0–34) µmol/l in the treatment group (*P* = 0.17) (Table 2 and Fig. 1). Worsening kidney function occurred in 20 (29%) control group patients and 3 (18%) treatment group patients (*P* = 0.54). New renal replacement therapy was initiated in 13 (19%) control group patients and 1 (6%) patient in the treatment group (*P* = 0.28) (Table 2). We observed higher daily urine output in the treatment group (*P* [interaction] < 0.001). We observed a pattern towards greater diuretic response to furosemide in the treatment group (*P* [interaction] = 0.08) (Fig. 3).

### Infectious outcome

Positive urine cultures were recorded in 9 (13%) control group patients and 4 (22%) treatment group patients (*P* = 0.28). Culture findings are presented in Additional file 1: Table S2.

### Exploratory clinical outcome

Overall, 14 (19%) control group patients and 3 (17%) treatment group patients died in hospital (*P* = 0.79) (Table 2).

## Discussion

### Key findings

We conducted a pilot case–control study in ICU patients with type 2 diabetes treated according to a liberal glucose control protocol to assess the relationship between empagliflozin therapy and renal, glycemic, metabolic, infectious, and exploratory clinical outcomes. We observed increasing blood sodium and chloride levels after empagliflozin initiation but no association with acid–base status. In keeping with such observations, the overall urinary output and diuretic response to furosemide appeared greater in the empagliflozin group. A numerically lower proportion of patients treated with empagliflozin developed worsening kidney function. In contrast, a positive urine culture was numerically more common after empagliflozin initiation. We observed no apparent relationship between empagliflozin treatment and hypoglycemia or ketoacidosis. Hospital mortality was approximately 20% in both groups.

### Relationship with previous studies

To the best of our knowledge, our study is the first to assess early physiological, biochemical, and microbiological responses to SGLT2 inhibitors in critically ill patients with type 2 diabetes. We observed a gradual increase in blood sodium levels after empagliflozin initiation. This is likely explained by free water loss from glucosuria-induced osmotic diuresis. Indeed, in a recent study, empagliflozin increased plasma sodium levels by 10 mmol/l on average after four days in patients with the syndrome of inappropriate antidiuresis [16]. A similar absolute increase was observed in our study. In addition, we observed an almost parallel increase in blood chloride levels and negligible differences in aSID, pH, and base excess trajectories compared to our matched control group. A lack of effect on acid–base status confirms data from previous animal experiments [17].

Monotherapy with SGLT2 inhibitors is not associated with increased risk of hypoglycemia compared with placebo [18]. In combination with insulin, this risk is significantly increased [19]. Despite a combination of insulin and empagliflozin therapy in the treatment group, we observed similar hypoglycemia occurrence (6%) in the two study groups. Our liberal glucose control protocol likely contributed to this low rate of hypoglycemia [14].

Ketosis is common in critically ill patients with type 2 diabetes on admission to the ICU but typically resolves within 24–48 h [15]. In the present study, empagliflozin treatment was not associated with greater ketotic burden and no patients developed ketoacidosis. Similarly, the occurrence of ketoacidosis was low (0.3%) among hospitalized COVID-19 patients randomized to dapagliflozin [7]. However, our limited sample size and the fact that most patients in the treatment group received insulin should be acknowledged. Additionally, the elimination half-life of β-hydroxybutyrate in healthy volunteers is only 1–3 h [20]. As we only measured β-hydroxybutyrate once daily around the time of empagliflozin administration, it is possible that we somewhat underestimated the prevalence of mild to marked ketosis. However, with accumulating doses, ketosis would likely be detected even with this infrequent sampling strategy.

Despite robust data on nephroprotective effects of long-term treatment with SGLT2 inhibitors, there are concerns that such therapy may lead to acute kidney injury due to reduced plasma volume, an early decline in glomerular pressure and GFR via tubulo-glomerular feedback activation, and hypoxia in the renal medulla [21–23]. However, recent studies refute these concerns. For example, in a meta-analysis of placebo-controlled trials, allocation to an SGLT2 inhibitor reduced the risk of AKI (defined by its specific Medical Dictionary for Regulatory Activities Preferred Term) by 23% (95% CI 19–26%) [1]. In a propensity score matched cohort study of elderly patients (mean age 72 years) with type 2 diabetes, initiation of an SGLT2 inhibitor was associated with reduced risk of hospitalization for AKI (discharge diagnosis) compared with dipeptidyl peptidase 4 inhibitors or glucagon-like peptide 1 receptor agonists [24]. In a Scandinavian cohort, new use of SGLT2 inhibitors, compared with dipeptidyl peptidase-4 inhibitors, was associated with reduced risk of serious renal events (RRT, death from renal causes, or hospitalization for renal events) (adjusted HR 0.42, 95% CI 0.34–0.53) [25]. In 1250 acutely ill patients with COVID-19, dapagliflozin, compared with placebo, resulted in numerically fewer events of worsening kidney function (doubling of serum creatinine or initiation of RRT) within 30 days (HR 0.65, 95% CI 0.38–1.10) [7]. In line with the above observations, our data suggest that treatment with empagliflozin is not associated with an early decline in kidney function in critically ill patients.

Evidence regarding risk of severe urinary tract infections with SGLT2 inhibitors are conflicting but most reports conclude that such events are rare [4]. Most ICU patients have an indwelling urinary catheter, which predisposes to asymptomatic bacteriuria. We observed a numerically higher rate of positive urine cultures in our empagliflozin treated patients but a similar rate of Candida albicans isolation. The clinical significance of this finding, however, is uncertain.

### Study implications

We have provided preliminary evidence of the effects and possible side effects of empagliflozin therapy in critically ill patients with type 2 diabetes exposed to permissive hyperglycemia. Our findings imply that empagliflozin likely increases free water clearance and increases loop-diuretic responsiveness. The clinical significance of the observed increase in sodium and chloride remains uncertain. If they increase in similar proportions, which appears to be the case, there would be no effect on acid base status. This is confirmed by our additional analysis of strong ion difference, base excess, and pH trajectories. Observational data suggest that hyperchloremia is associated with acute kidney injury development [26]. This is not supported by our data, which showed a numerically lower incidence of worsening kidney function in the treatment group. Yet, clinicians should be aware of the effect of empagliflozin on free water clearance and corresponding changes in sodium and chloride and should consider minimizing the content of these solutes in administered fluids. Within the limitations of small numbers, our findings also imply that empagliflozin therapy in the ICU might reduce insulin requirements without inducing ketoacidosis. The majority (> 80%) of treatment group patients received insulin at the time of empagliflozin initiation. As insulin suppresses lipolysis and ketogenesis, early ketoacidosis was not expected in this group. However, our data implies that continuing empagliflozin therapy may not lead to delayed ketoacidosis despite gradual weaning from insulin therapy. At least during exposure to permissive hyperglycemia, the combination of empagliflozin and insulin does not appear to increase the risk of hypoglycemia. Furthermore, our results do not support the view that SGLT2 inhibitors result in acutely decreased kidney function. Finally, our data support further cautious assessment of the safety and efficacy of SGLT2 inhibitors in the ICU setting in randomized controlled trials.

### Strengths and limitations

Our study has limitations. It is not a randomized controlled trial. Furthermore, the treating staff was unblinded to β-hydroxybutyrate levels and empagliflozin treatment. This could potentially have biased insulin dosing decisions. Although cases and controls were matched on age, HbA1c, and ICU length of stay, they differed significantly with respect to other baseline characteristics. For example, treatment group patients were less likely to be admitted from the operating room and more likely to be admitted from the emergency department, representing subgroups with different risk of ketoacidosis development. The limited sample size prevented adjustment of potential confounders in the analyses. However, our exploratory analysis is an important first step to inform and justify the cautious step-by-step conduct and design of future trials. Patients were exposed to a liberal glucose control strategy. Our findings should, therefore, not be generalized to ICU patients managed according to more strict blood glucose control protocols.

## Conclusions

In our exploratory pilot cohort of ICU patients with type 2 diabetes managed according to a liberal glucose control protocol, treatment with empagliflozin reduced insulin requirements and was associated with a significantly greater increase in sodium and chloride levels and urinary output. Empagliflozin therapy was not associated with hypoglycemia, ketoacidosis, worsening kidney function, or a statistically significant increase in bacteriuria. These findings provide the basis for further cautious investigation of this intervention in critically ill patients.

## Supplementary Information

**Additional file 1: Table S1.** Pre-ICU diabetes therapy in treatment group patients. **Table S2.** Urine culture findings in the control group and treatment group, respectively. **Fig. S1.** Liberal protocol for blood glucose management of patients with diabetes at the Austin Hospital Intensive Care Unit. **Fig. S2.** Distribution of blood ketone measurements with respect to sample time in control group patients and treatment group patients.

## Data Availability

The data that support the findings of this study are available from the authors upon reasonable request.

## Abbreviations

AKI: Acute kidney injury
aSID: Abbreviated strong ion difference
CI: Confidence interval
COVID-19: Coronavirus disease-19
GFR: Glomerular filtration rate
HbA1c: Glycated hemoglobin A1c
HR: Hazard ratio
ICU: Intensive care unit
IQR: Interquartile range
RRT: Renal replacement therapy
SGLT2: Sodium glucose co-transporter-2
