# Supplementary material for: Sodium glucose co-transporter-2 inhibitors in intensive care unit patients with type 2 diabetes: a pilot case control study
Source: Crit Care. 2023 May 16;27:189. doi: 10.1186/s13054-023-04481-y (PMC10186281; doi:10.1186/s13054-023-04481-y)
Supplement: Supplementary file 1 — Additional file 1: Table S1. Pre-ICU diabetes therapy in treatment group patients. Table S2. Urine culture findings in the control group and treatment group, respectively. Fig. S1. Liberal protocol for blood glucose management of patients with diabetes at the Austin Hospital Intensive Care Unit. Fig. S2. Distribution of blood ketone measurements with respect to sample time in control group patients and treatment group patients. [file 13054_2023_4481_MOESM1_ESM.pdf]

Additional File 1

Table S1. Pre-ICU diabetes therapy in treatment group patients

Diabetes therapy	Treatment group (n = 18)
**Insulin only**	**4 (22.2%)**
**Insulin & noninsulin glucose-lowering drugs**	**4 (22.2%)**
Insulin + Acarbose	1 (5.6%)
Insulin + Biguanide + SGLT2 inhibitor (other than empagliflozin)	2 (11.1%)
Insulin + Sulfonylurea	1 (5.6%)
**Noninsulin glucose-lowering drugs only**	**8 (44.4%)**
Biguanide	2 (11.1%)
Biguanide + Sulfonylurea	1 (5.6%)
Biguanide + Sulfonylurea + DDP4 inhibitor	3 (16.7%)
Biguanide + Sulfonylurea + SGLT2 inhibitor (other than empagliflozin)	1 (5.6%)
Biguanide + DDP4 inhibitor	1 (5.6%)
**Diet control only**	**2 (11.1%)**
Abbreviations: SGLT2, Sodium glucose co-transporter-2 inhibitor; DDP4, Dipeptidyl peptidase-4 inhibitor	

Table S2. Urine culture findings

Variable	Control group (n=72)	Treatment group (n=18)
Positive urine culture (any pathogen)	9/72 (13%)	4/18 (22%)
Urine culture pathogen[a]		
Pseudomonas aeruginosa	1	1
Yeast	0	2
Candida albicans	5	1
Staphylococcus haemolyticus	1	0
Enterococcus faecalis	2	0
Enterobacter aerogenes	1	0
Klebsiella pneumoniae	1	0
[a]More than one pathogen was found in some cultures		

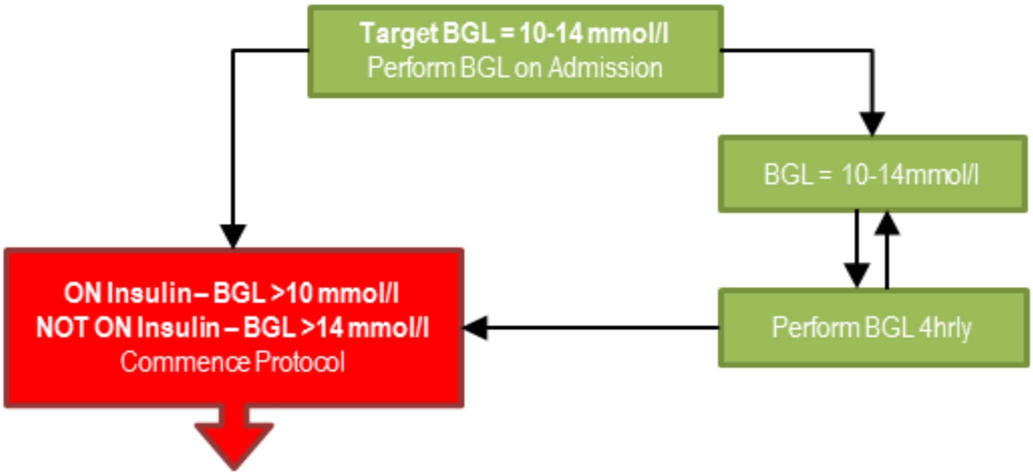

Table: Insulin Infusion Protocol

BGL	Bolus	Starting Infusion	Subsequent Infusion	Repeat BGL
mmol/l	Units IV	Units/hr	Units/hr	Hours
>20	6	BPIR	Increase by 2	1
>16	4	BPIR-0.5	Increase by 1	1
>14	2	BPIR-1	Increase by 1	1
10-14	0	BPIR-1.5	Continue current rate	1
8-10	0	0	Drop by 1	1
6-8	0	0	Drop by 2	1
4-6	0	0	Stop	1
<4	0	0	Stop and administer 10ml of 50% glucose at 1 ml/min	1

Figure S1. Liberal protocol for blood glucose management of patients with diabetes at the Austin Hospital Intensive Care Unit since 2015. Baseline pre-morbid insulin requirements **(BPIR)** is for diabetic patients who are previously receiving insulin and is the total insulin that the patient is receiving prior to their acute illness divided by 24 (hours).

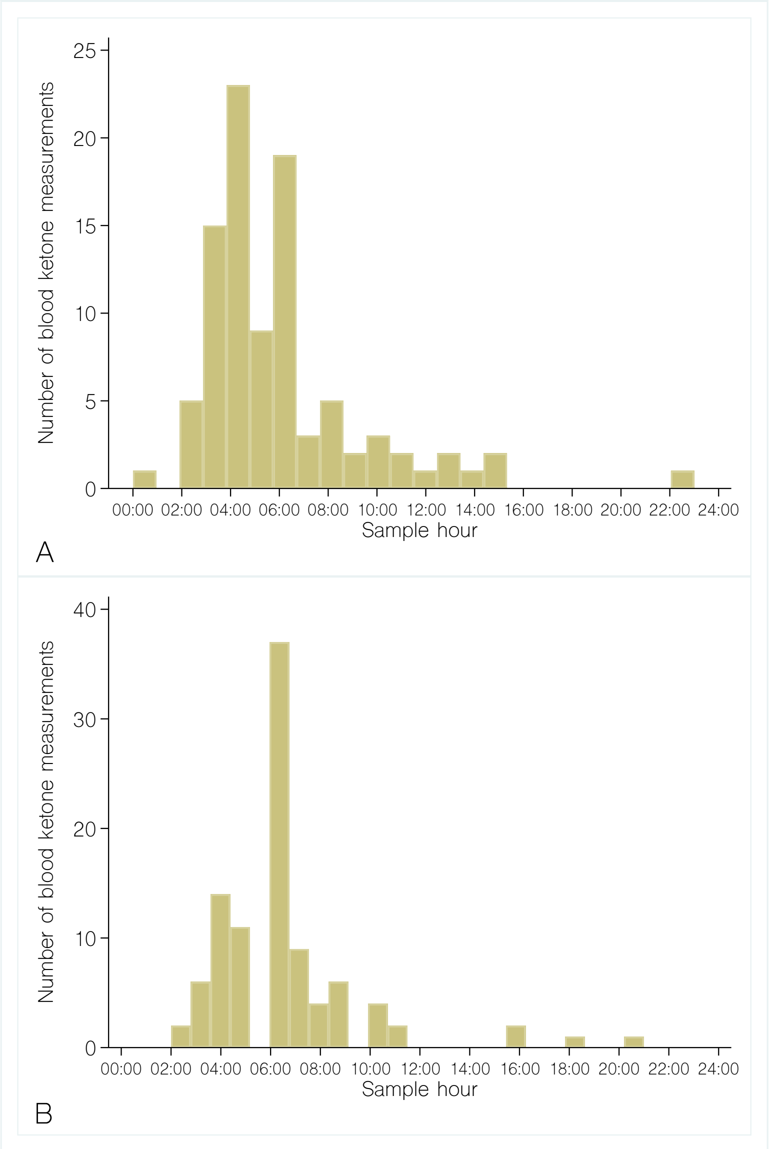

Figure S2. Distribution of blood ketone measurements with respect to sample time in control group patients (A) and treatment group patients (B).
